# Effects of selenium-enriched *Bacillus* sp. compounds on growth performance, antioxidant status, and lipid parameters breast meat quality of Chinese Huainan partridge chicks in winter cold stress

**DOI:** 10.1186/s12944-019-1015-6

**Published:** 2019-03-14

**Authors:** Jiajun Yang, Minhong Zhang, Ying Zhou

**Affiliations:** 1 State Key Laboratory of Animal Nutrition, Institute of Animal Sciences, Chinese Academy of Agricultural Sciences, Yuanminyuan West Road, Haidian District, Beijing, 100094 China; 2 Anhui Province Key Laboratory of Livestock and Poultry Product Safety Engineering, Institute of Animal Husbandry and Veterinary Medicine, Anhui Academy of Agriculture Science, NongKe South of Road, Hefei, 230031 Anhui China

**Keywords:** Selenium-enriched *Bacillus*, Chick, Growth performance, Breast meat, Stress

## Abstract

**Background:**

Both selenium (Se) and probiotic *Bacillus* regulate the metabolism to help defense clod stress and improve the meat quality in breeding chicks. The purpose of this study was to evaluate the effect of supplemental Se and *Bacillus* in the form of Se-enriched *Bacillus* (SECB) on the growth performance, lipid parameters, breast Se and antibiotic levels, and breast meat quality of chicken in winter cold stress.

**Methods:**

Five hundred 1-d-old chickens were divided into five groups randomly: Control, inorganic Se, compound *Bacillus*, SECB, and antibiotic. The feed duration was 56 d.

**Results:**

After 28 d of treatment, chicks feed SECB or compound *Bacillus* had higher body weights than the control, and after 56 d, chicks given either SECB or compound *Bacillus* had higher body weights than the control chicks or those given inorganic Se. Adding SECB to feed significantly increased the lightness, redness, and yellowness of breast meat, improved the water-holding capacity, and reduced the shear force and cooking loss. The concentration of Se in the breast muscle very significantly increased after SECB and inorganic Se supplementation, which was opposite to the concentration of flavomycin in antibiotic supplemented chicks. The antioxidative status of plasma and breast meat was significantly improved with added compound *Bacillus* and SECB: the total antioxidant capacity, total superoxide dismutase, and glutathione peroxidase ability in the breast muscle significantly improved, and the malondialdehyde concentration in plasma decreased. The levels of total cholesterol plasma triglyceride and very-low-density lipoprotein cholesterol in the plasma and breast muscle was decreased compared to that of the control, while the plasma high-density lipoprotein cholesterol concentration increased.

**Conclusions:**

In conclusion, SECB supplementation promoted the body growth, antioxidative status, and Se concentrations in the plasma and breast meat, and also improved the breast meat quality.

## Background

In the winter of most part of China, the outside temperature could be below 0 °C. Usually, the temperature is below the suitable living standard for chick growth; it can lead to cold stress and diseases, causing bad meat quality and low breeding efficiency.

Selenium (Se) is an important trace element; its beneficial effects are well documented [1] which is relatively deficient in most areas of the world. Dietary Se deficiency is considered to be a causative factor of abnormal myocardial matrix remodeling and dysfunction in the normal heart [2]. Suitable doses of Se supplementation can regulate the metabolism of nutrient substances and improve digestion, stress, and antioxidation in animals [3–5]. Supplementation of the diet with tetravalent Se (IV) can be achieved using a salt of selenium, such as sodium selenite. Low-molecular-weight organic Se complexes, such as amino acids [6] and nano forms [7], provide a myriad of benefits; they have a higher organic bioavailability than the inorganic forms that are most often used as a dietary supplement [8]. However, their higher cost may inhibit widespread use. There are a number of stressors in industrial chick production, such as heat, cold, and group-transferred [9]. In order to defend against stress, other nutritional indigents would be assigned, which is not beneficial to body growth and meat quality. Considering the anti-stress role of Se, it is of urgent need to explore a cheap and convenient organic Se source for supplementation.

Probiotics contain live microorganisms and spores which when administered in adequate amount, confer health benefts to the host [10]. *Bacillus subtilis* (*B. subtilis*) and *Bacillus licheniformis* (*B. licheniformis*) are the two most widely used strains of probiotic bacteria in animal diets [11, 12]. Oral administration of *B. subtilis* and *B. licheniformis* can have a myriad of beneficial effects, such as improved growth and meat characteristics [13–15], optimized composition of intestinal microbiota, prevention of some diarrheal diseases [16], and reduced stresses [17]. For such benefits, *B. subtilis* and *B. licheniformis* have attracted considerable attention as a potentially beneficial dietary supplement for animal health.

Considering the positive effects of Se and *B. subtilis* and *B. licheniformis* on growth performance, anti-oxidation, and stress [17, 18], it would be valuable to study whether the combined use of Se and compound *Bacillus* has greater effects on body growth and meat quality.

For the many negative effects of antibiotic drugs used in chick production, we want to compare the positive effects of two strains of compound *Bacillus* spp. and SECB to those of antibiotic drugs, which would be alternatively used in chick production. Chinese Huainan Partridge chicken is a dual-purpose native breed in South China. Because of the special habits of consuming, South Chinese are interested in selecting the breed; hence the need for this study [19].

To test this hypothesis, we produced Se-enriched *B. subtilis* (SECB), which combines the virtues of *B. subtilis* and those of organic Se, and might induce an enhanced defense response against cold stress and improved meat quality through dietary supplementation.

## Methods

### Chicks and management

The animal treatment, housing, and husbandry conditions conformed to the experimental guidelines of the Institutional Animal Care and Use Committee of China. The experimental protocols in this study, including animal husbandry and slaughter, were approved by the Institution of Animal Science and Welfare of Anhui Province (no. IASWAP2017110649).

A total of 500 one-d-old Chinese Huainan Partridge chicken (average body weight, 40.15 g) were randomly allocated into five groups with six replicates of 25 each. Chickens in the control group were fed a basal diet and the four treatments were fed the following: basal diet with either inorganic sodium selenite (IS), compound *Bacillus* (CB), selenium enriched compound *Bacillus* (SECB) and flavomycin. Experimental diets were fed in two periods: starter (days 0–28) and finisher (days 29–56). The composition and nutrient analysis results for the basal diet, which did not contain any probiotics or antibiotics, are shown in Table 1. All the nutrients met or exceeded the nutrient requirements (National Research Council, 2012) [20]. The chicks were net-reared from November 22 of 2017 to January 16 of 2018. For the duration of the starter diet, the pen space for each replicate was 2.3 m^2^. The room temperature was kept at 33–35 °C by an electric heating tube in the first week, then gradually declined to 21 °C at the end of the fourth week. At the end of the starter diet period, all chicks were weighted and transferred into larger pens (5.6 m^2^ for each replicate); chicks in the previous replicate were also transferred and kept under the same conditions. In the fifth week, the temperature was maintained at 18–21 °C by infrared warming lights. In weeks six to eight, the number of infrared warming lights was reduced gradually for a room temperature of 8.5–15.5 °C. The chickens were allowed ad libitum access to water and feed throughout the experimental periods. The normal immune procedure was implemented throughout the whole trial.

**Table 1:** Composition and nutrient analysis of the basic diet for broilers at different stages

	Ingredient	Starter (0~28) %	Finisher (29~56) %
Item	Corn	57.97	61.75
	Soybean meal	29.30	26.45
	Fish powder	5.00	3.51
	Soybean oil	2.00	3.00
	Premix	5.00^a^	5.00^a^
	Dicalcium phosphorus	0.47	0.29
	Limestone	0.26	0
Calculated nutrient	Metabolizable energy (MJ /kg)	12.12	12.54
	CP	21	19
	Calcium	1	0.9
	Total phosphorus	0.68	0.65
	Available phosphorus	0.45	0.38
	Lys	1.05	0.9
	Met	0.46	0.3

The premix provides

^a^vitamins and trace elements per kg diet: Vitamin A (retinyl acetate) 9, 875 IU, Vitamin D_3_ (cholecalciferol) 3, 000 IU, Vitamin E (DL-ɑ-tocopheryl acetate) 20 IU, menadione 3.25 mg, Vitamin B_12_ (cyanocobalamin) 0.025 mg, thiamin 1.5 mg, riboflavin 5.0 mg, biotin 0.0 32 mg, folacin 1.25 mg, niacin 12 mg, pantothenic acid 12 mg, and pyridoxine 3.75 mg, manganese 100 mg, zinc 80 mg, iron 80 mg, copper 8 mg, iodine 0.15 mg, and selenium 0.15 mg

### Feed for each group

*B. subtilis* and *B. licheniformis* were isolated from the ileum of a healthy Chinese Huainan Partridge chicken by a group researching probiotic bacteria, the Institute of Animal Husbandry and Veterinary Medicine, Anhui Academy of Agricultural Sciences, which were stored in the China General Microbiological Culture Collection Center. We cultured *B. subtilis* and *B. licheniformis* with a liquid beef extract–peptone medium. The number of live *Bacillus* reached 6.6 × 10^8^ CFU/mL, respectively, after fermentation. The fermentation of selenium-enriched *B. subtilis* and *B. licheniformis* was prepared with sodium selenite supplemented into the culture medium until the Se concentration reached 50 μg/mL. After fermentation, SECB were harvested, the organic Se reached 48.13 μg/mL mainly as Se protein (contained 93.17%), and live numbers of *B. subtilis* and *B. licheniformis* were 3.3 × 10^7^ and 6.6 × 10^7^ CFU/mL respectively. For the chick in the IS group, 1.12 g of sodium selenite (analytically pure) was diluted into 100 mL of distilled water, which was blended with 5 kg of feed. Then, the mixed mass feed was added into a blender contained 90 kg of mass feed. The blender was employed for 20 min to mix the additives uniformly. The feed for the flavomycin group was prepared using 4 g of premixed food containing 10% flavomycin, which was blended with 100 kg of feed, to reach a concentration of 4 mg/kg. For compound *Bacillus*, 50 mL of *B. subtilis* and 100 mL of *B. licheniformis* fermentation liquid were measured separately and first blended with 5 kg of feed, and then with 95 kg of mass feed after. The SECB feed was prepared by blending 1000 mL of SECB fermentation liquid with 100 kg of feed. After preparing the five different feedstuffs, the population of *B. subtilis* and *B. licheniformis* were counted using the plate method with a yeast extract peptone dextrose medium. The concentration of Se in all feed types was also measured. The results were listed in Table 2.

**Table 2:** Concentration of Se and number of *B. suthilis* in Groups

Groups	Concentration of Se (ng/g)	number of *B. suthilis*:*B.licheniformis* (CFU/g)
Control	102.0	0
IS	602.0	0
* CB*	102.0	3.3 × 10^5^:6.6 × 10^5^
SECB	602.0	3.3 × 10^5^:6.6 × 10^5^
Flavomycin	102	0

### Performance and sample collection

Chicks in every replicate of each treatment group were weighed on 0 d, 28 d, and 56 d. Daily feed consumption was accurately recorded. Daily weight gain and ratio of feed to gain (F:G) were calculated. ADG = body increase (g)/number of days. F:G ratio = mass of food intake (g)/body increase (g).

After 56 d, two chickens from each replicate were selected, fasted for 12 h, and then the tissue and blood were harvested under general halothane anesthesia. All blood samples were collected in 5.0 mL sterile heparinized tubes. We removed 1 mL of each blood sample to measure the Se concentration. Then, remnant chick blood was centrifuged at 3000 rpm/min for 10 min to collect the plasma for biochemical assays (described below). Breast meat was collected and stored at 4 °C until analysis.

### The assay of antioxidation

The blood of chickens was harvested from the wing vein and precipitated at 3000×g for 10 min to obtain the plasma. Then, 0.3 g of breast muscle samples in each group was weighed to prepare the homogenate. Plasma and breast muscle malondialdehyde, anti-oxidative enzymes (superoxide dismutase, glutathione peroxidase), and total antioxidant capacity were detected using kits. Six replicates were used in each experiment.

### Lipid parameters analyses

Concentration of total cholesterol in the plasma and breast muscle, plasma triglyceride and very low- and high-density lipoprotein cholesterol concentrations were measured using the appropriate detection kits (Nanjing Jiancheng Bioengineering Institute).

### Breast meat quality analysis

Breast muscle samples were collected for meat quality at the end of the experiment. After being maintained at 4 °C for 24 h, the meat redness (a*), yellowness (b*), and lightness (L*) was determined by a Chroma meter (CR-410; Minolta Co., Tokyo, Japan). Water-holding capacity (WHC) was measured by determining expressible juice using a modification of the filter paper press method indicated by Wierbicki and Deatherage, as follows [21]. The breast meat was refrigerated overnight at 4 °C and then brought to room temperature before cooking to measure the cooking loss and shear force. The breast muscle from each bird was placed into a thin walled plastic bag and cooked to an internal temperature of 70 °C in a water bath with a digital thermostat. The cooked muscle was cooled to room temperature. The Warner–Bratzler shear force was determined using an Instron Universal Mechanical Machine (Instron Model 4411; Instron Corp., Canton, MA). Each sample was then cooled under tap water and equilibrated at room temperature. The muscle was weighed again for determination of cooking loss (%). Shear force was inspected by cutting eight 1 × 1 cm cores of about 3 cm thick, harvested parallel to the fiber orientation, through the thickest portion of the cooked muscle.

### The assay of se concentration

A ZEEnit 700 P atomic absorption spectrometer (Analytik Jena, Germany) was employed to determine the selenium levels in the breast meat and plasma. All measurements were performed using the described method [22]. First, 0.3 g samples of breast meat and 1.0 mL of plasma from all groups were placed in beakers and digested by adding 10 ml of a nitric acid–perchloric acid (HNO3–HClO4) mixture. The mixture was heated on a sand bath until fumes appeared (the temperature was maintained at 200 °C) and the solution had mostly evaporated. After cooling, 5 mL of hydrochloric acid solution (6 mol/L) was added, and the heating procedure was repeated at 180 °C. The cooled mixture was made up to 10 mL with 5% hydrochloric acid solution. Eight replicates were used for each group.

### Flavomycin assay

Samples of 0.3 g of breast meat were weighed and placed in 5 mL plastic tubes. Then, 3.6 mL of ammonium methanol (ammonia:methanol [v:v] = 9:1) were added to homogenize. The homogenous liquid was centrifuged at 12,000×g for 3 min at 4 °C. After, 0.5 mL of supernatant was removed into a sterilized plastic tube and 2 mL acetonitrile was added, which were precipitated at 12000×g for 10 min at 4 °C. The supernatant was removed into another sterilized plastic tube, and dried using a stream of nitrogen. The samples were made up to a volume of 1 mL using methanol, and filtered using 0.22-μm filter membrane. The concentration of flavomycin was measured with liquid chromatography–tandem mass spectrometry (LC-MS/MS) following the methods of the reported reference [23].

### Statistical analyses

Body weight, meat quality, Se and flavomycin concentration, and antioxidation data were subjected to one-way ANOVA using the GLM procedure of SPSS, with significance reported at *P* < 0.05. Means were further separated using Duncan’s multiple range test. Data on body weight, average daily gain, average daily feed intake, and F:G ratio were statistically processed as repeated measures. A *P*-value of less than 0.05 was considered statistically significant.

## Results

### Growth performance

**Fig. 1:**
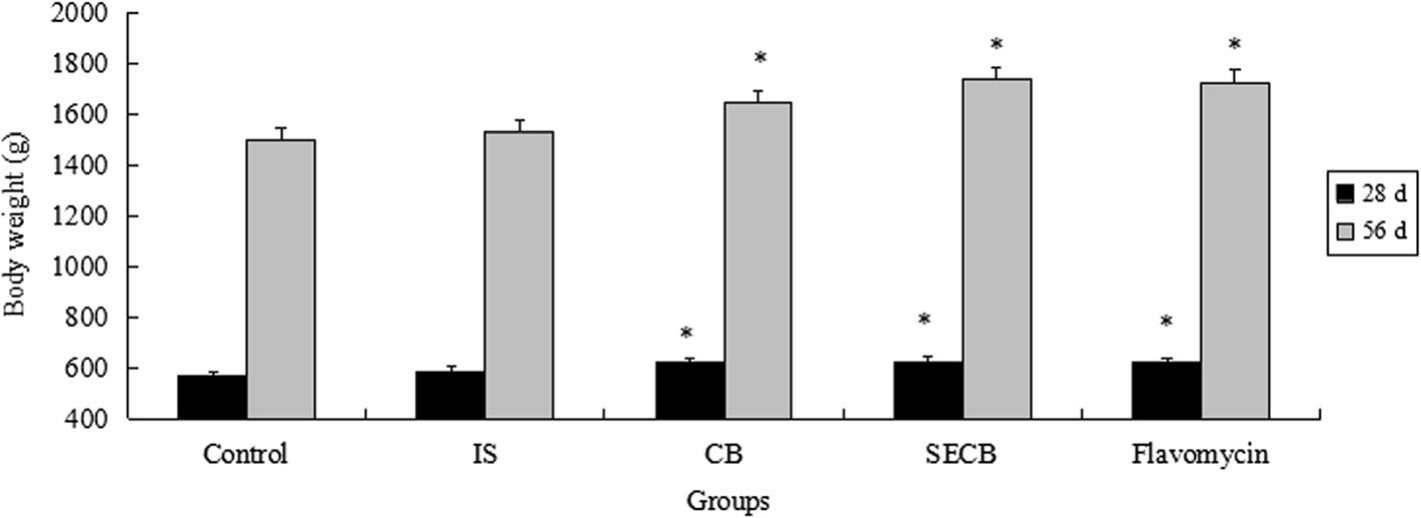
Effects of different treatments on chicken body weight. The chicken were treated with control, inorganic Se (IS), compound Bacillus (CB), selenium enriched compound Bacillus (SECB) and antibiotic (flacomycin) after 28 and 56 days. Bars represent mean ± S.E. Bar in same color with with”*” is significantly different from those without (*P* < 0.05)

**Fig. 2:**
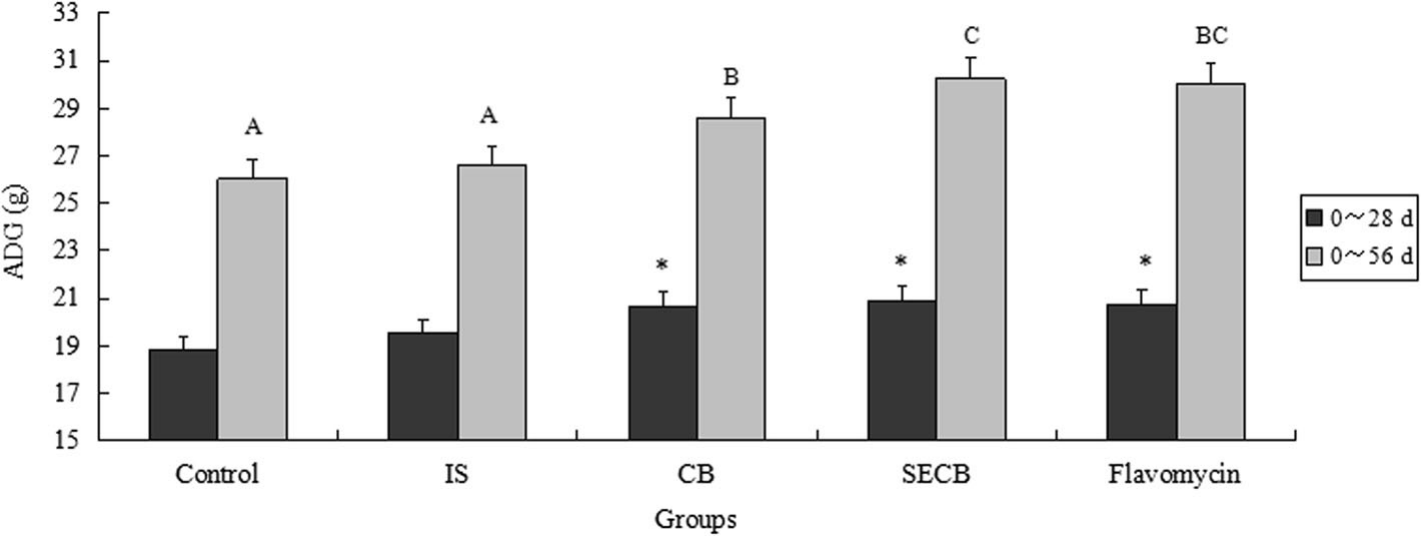
Effects of different treatments on chicken average daily gain. The chicken were treated with control, IS, CB, SECB and flacomycin after 28 and 56 days. Bars represent mean ± S.E. Bar in same color with with A, B mean significant difference at 0.01 levels (*P* < 0.01)

**Fig. 3:**
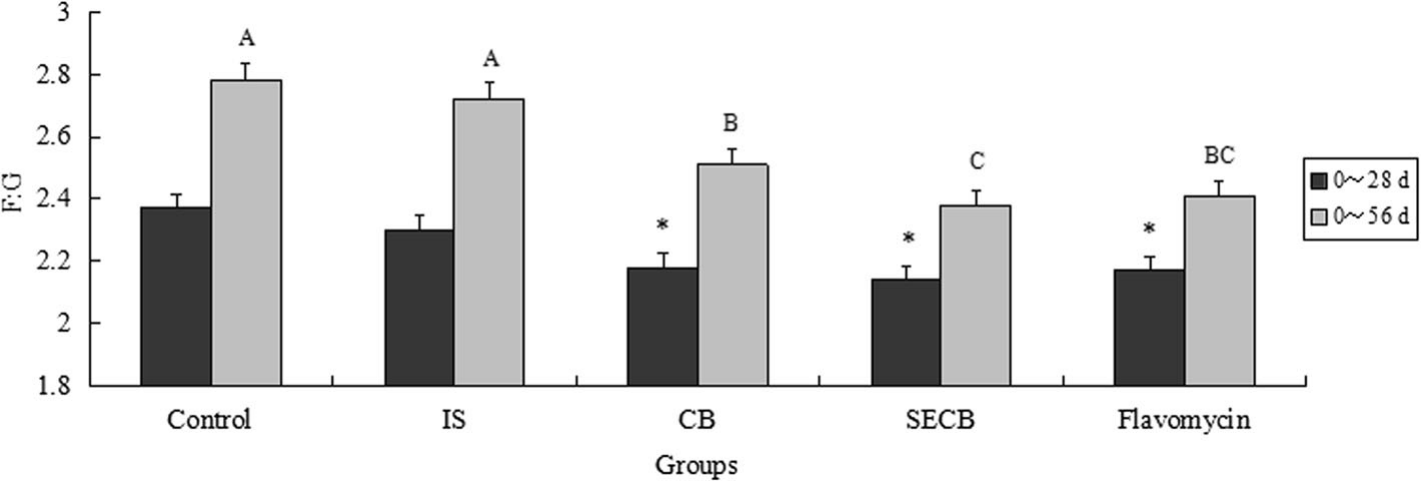
Effects of different treatments on chicken ratio of feed to gain. The chicken were treated with control, IS, CB, SECB and flacomycin after 28 and 56 days. Bars represent mean ± S.E. Bar in same color with A, B, C mean significant difference at 0.01 levels (*P* < 0.01)

The growth performance of Chinese Huainan Partridge chickens in five groups is shown in Figs. 1, 2 and 3. At 28 d, chickens in groups supplemented with CB, SECB, and flavomycin had higher final body weights than those in the control or IS groups (*P* < 0.05). The ADG indices of the control and IS groups were not significant (*P* > 0.05) at 28 d, and were significantly lower than those of the CB, SECB, and flavomycin supplemented groups (*P* < 0.05). However, the result of the F:G ratio was opposite to that of the final body weight. Birds that received CB, SECB, and flavomycin had lower F:G ratios (*P* < 0.05). There were no differences in the ADFI among the four groups either after 28 or 56 d (*P* > 0.05).

After 56 d, the final body weights in the CB, SECB and flavomycin groups were still significantly higher compared to those of the control (about 235 g) and inorganic Se-supplemented groups (*P* < 0.05). The ADG indices of the CB, SECB and flavomycin groups were significantly higher than those of the control and IS groups (*P* < 0.01). There were no differences in ADG between the CB and flavomycin or flavomycin and SECB groups (*P* > 0.05). Over the entire feeding duration, the F:G ratio in the SECB group was the lowest (*P* < 0.05). The F:G ratio of SECB was the lowest among all groups (*P* < 0.01), but had no significant difference to that of the flavomycin group (*P* > 0.05). The indices of chicks in the CB and flavomycin supplemented groups declined very significantly compared to those of chicks in the control and IS groups (*P* < 0.01).

### Antioxidative levels of plasma and breast meat

After 56 d, the antioxidation capacity of plasma and breast meat was analyzed (Table 3). The concentrations of MDA in the plasma of all supplemented groups declined significantly compared to that of the control. The MDA content in the SECB group was the lowest, at 3.21 μmol/L lower than that of the control (*P* < 0.05). The MDA concentration of breast muscle in all supplementary groups was lower than that of the control (*P* < 0.05), and that of SECB was the lowest of all groups (*P* < 0.05). There were no differences among the other supplemented groups (*P* > 0.05). The superoxide dismutase (SOD) activity results were in reverse to the concentration of MDA results. The SOD activity was increased in both the plasma and breast muscle in all supplementary groups (*P* < 0.05), while the index in SECB was the highest among all five groups (*P* < 0.05). No significant differences were found among the IS, CB, and flavomycin groups (*P* > 0.05). The activities of glutathione peroxidase (GPx) in the two groups with Se supplementation were significantly higher than those in all other groups (*P* < 0.05) both in plasma and breast muscle. The activity of GPx in plasma with added SECB was higher than that of the control, with approximately 44.79 and 27.58 units in the plasma and breast muscle, respectively. There were no differences among the groups without Se supplementation (*P* > 0.05). The total antioxidant capacity (T-AOC) index in the control was lower than those in other treatments, both in the plasma and breast muscle (*P* < 0.05). There were no differences in the T-AOC of plasma among all groups (*P* > 0.05). In the breast muscle, the T-AOC result of the SECB group was higher than that of the IS and flavomycin groups (*P* < 0.05), and no difference was found in the CB group (*P* > 0.05).

**Table 3:** Effects of different treatments on antioxidation of plasma and breast muscle

Groups	MDA μmol·L^−1^	SOD U/mL	GPX mol/L	T-AOC Unit / mL serum
	Plasma			
Control	8.47^a^	101.05^a^	318.45^a^	13.96^a^
IS	7.53^b^	111.24^b^	359.37^b^	14.83^b^
CB	6.49^c^	115.36^b^	319.51^a^	14.98^b^
SECB	5.25^d^	130.45^c^	363.24^b^	15.25^b^
Flavomycin	6.53^c^	110.15^b^	312.33^a^	14.97^b^
SEM	0.06	1.25	3.94	0.11
*P*-value	0.14	0.15	0.16	0.18
	Breast muscle			
Control	10.02^a^	83.22^a^	192.31^a^	11.56^a^
IS	8.89^b^	96.51^b^	220.26^b^	12.63^b^
CB	7.92^b^	101.92^b^	195.52^a^	13.18^bc^
SECB	6.05^c^	110.39^c^	219.89^b^	13.45^c^
Flavomycin	8.54^b^	102.61^b^	188.98^a^	12.67^b^
SEM	0.07	1.19	2.01	0.09
*P*-value	0.09	0.07	0.10	0.11

The different superscript small letters in the same column a,b mean significant difference at 0.05 levels (*P* < 0.05)

### Lipid parameters analyses

**Fig. 4:**
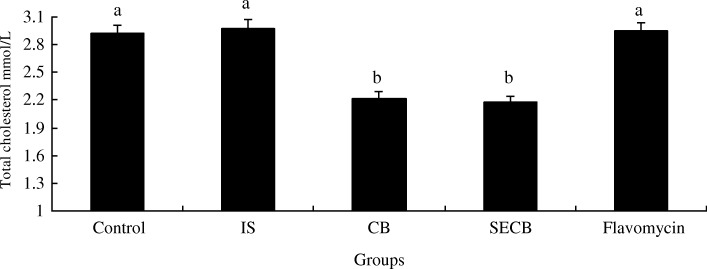
Effects of different treatments on total cholesterol of breast muscle. The chicken were treated with control, IS, CB, SECB and flacomycin after 28 and 56 days. Bars represent mean ± S.E. Bar in same color with a, b is significantly different from those without (*P* < 0.05)

The levels of (Table 4) total cholesterol, triglyceride, and very low- and high-density lipoprotein cholesterol in the SECB group were significantly lower than those in the control (*P* < 0.05), but were not significantly different to those in the CB group (*P > 0.05*). The amounts of HDL in the plasma in the SECB and CB groups were higher than that in the control group (*P* < 0.05). The concentration of total cholesterol (Fig. 4) in the SECB group was 0.75 mmol/L lower than that of the control group (*P* < 0.05), which showed difference to that of the CB group (*P* > 0.05).

**Table 4:** Effects of different treatments on lipid parameters of plasma

Groups	TC mmol/L	TG mmol/L	LDLC mmol/L	HDLC mmol/L
	Plasma			
Control	3.61^a^	1.05^a^	1.51^a^	0.71^a^
IS	3.63^a^	1.04^a^	1.48^a^	0.73^a^
CB	3.19^b^	0.91^b^	1.28^b^	0.83^b^
SECB	3.11^b^	0.88^b^	1.24^b^	0.85^b^
Flavomycin	3.58^a^	1.06^a^	1.53^a^	0.75^a^
SEM	0.08	0.03	0.05	0.02
*P*-value	0.14	0.11	0.16	0.09

The different superscript small letters in the same column a,b mean significant difference at 0.05 levels (*P* < 0.05)

### Breast meat quality

The breast meat color (L*, a*, and b*), water-holding capacity, shear force, and cooking loss were measured on 56-d-old chicks, and the results are shown in Table 5. Chicks in the two CB supplemented groups had lower L* and b*, and higher a* values (*P* < 0.05) than chickens in the control, IS, and flavomycin groups (*P* < 0.05). The L*, a*, and b* indices of chicks supplemented with CEBS were optimal in all treatments (*P* < 0.05). Birds supplemented with SECB and CB had higher water-holding capacities and lower cooking losses than those of the control group (*P <* 0.05). In addition, the index of cooking loss in the SECB group was the lowest of all the groups (*P* < 0.05). The results of shear force in the four treatments were lower than that of the control (*P* < 0.05), while the result of the SECB group was the lowest of all the groups (*P* < 0.05).

**Table 5:** Effects of different treatments on chicken breast meat characteristics

Groups	Meat color			WHC^4^ (%)	Shear force (kg/mm^2^)	Cooking loss (%)
	L^*^	a^*^	b^*^			
Control	51.18^a^	12.71^a^	16.02^a^	66.47^a^	2.81^a^	16.95^a^
IS	52.36^a^	12.93^a^	16.84^a^	67.91^a^	2.76^a^	15.99^ab^
CB	49.19^b^	13.21^b^	15.22^b^	72.17^b^	2.23^b^	14.81^b^
SECB	46.02^c^	15.38^c^	14.08^c^	72.98^c^	1.90^c^	13.11^c^
Flavomycin	51.27^a^	12.36^a^	16.11^a^	66.79^a^	2.79^a^	16.45^a^
SEM	0.45	0.14	0.16	0.48	0.03	0.10
* P-value*	0.031	0.026	0.021	0.019	0.028	0.034

The different superscript small letters in the same column a,b mean significant difference at 0.05 levels (*P* < 0.05)

### Levels of selenium and flavomycin

**Fig. 5:**
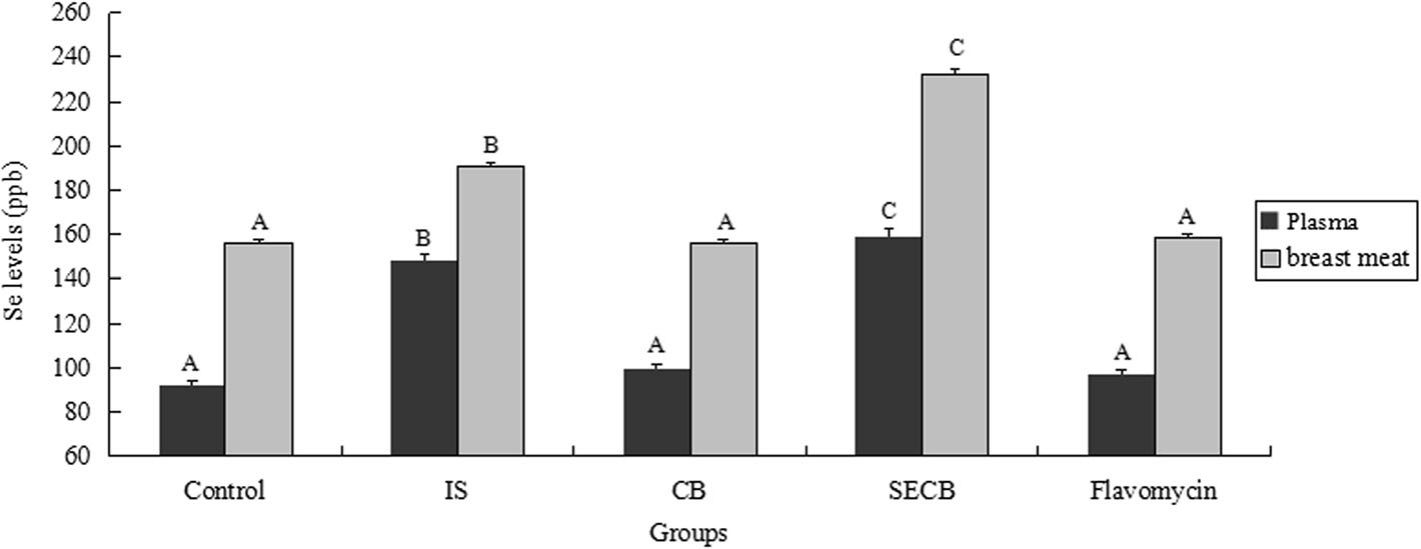
Effects of different treatments on selenium levels of plasma and breast meat. The chicken were treated with control, IS, CB, SECB and flacomycin after 28 and 56 days. Bars represent mean ± S.E.Bar in same color with different small letters A, B, C mean significant difference at 0.01 levels (*P* < 0.01)

The Se levels in the plasma and breast meat were measured, with the results shown in Fig. 5. The results indicated that the IS group had significantly more Se in the plasma and breast meat than the control and CB supplementation groups (*P* < 0.01). Chickens in the SECB group had the highest Se levels in both tissues (*P* < 0.01).

The flavomycin concentration of breast muscle in supplemented groups reached 76.13 ng/g meat, while there were no detected results in non-supplemented groups.

## Discussion

The merits of Se and probiotic bacteria on body were well-documented [19, 24]. *B. licheniformis* and *B. subtilis*, two strains of probiotic *Bacillus*, have a better ability to live in adverse environments (low-moisture, high pelleting temperature, and less nutrients) than *Lactobacillus*, [25], which were more widely developed as additives in the biomedical industry. SECB owing the organic Se and compound *Bacillus* (*B. licheniformis* and *B. subtilis*) would combine the beneficial effects of the two factors on growth performance of chick. In this research, the results indicated that with CB and SECB supplementation, chicks have higher body weights when transferred at 28 d. While there were no significant effects of IS supplementation. The promoting effect on growth was also shown in the final body weight (56 d) of the CB, SECB, and flavomycin groups. Both Se and *B. subtilis* have the ability to modulate the growth performance of chickens [18, 26, 27]. In our study, we supplemented basal feedstuff with a dose of 0.5 μg/g Se in an inorganic form, which had no positive effects on Chinese Huainan Partridge chicken; this is in accordance with existing studies [28, 29]. The results indicated that growth rates were improved in chickens given CB or SECB supplements. The microbial metabolites include antimicrobial substances like iturin and surfactin produced by *B. subtilis*, and enzyme protease, lipase, and amylase produced by *B. licheniformis*, which play an important role in keeping the body healthy and breaking down feed for nutrient absorption [30]. Chickens supplemented with SECB had (on average) higher body weights and greater feed utilization efficiencies than did the control group chickens. The F:G ratio of the SECB group was the lowest over the entire feeding period, suggesting that this treatment was more efficient than CB alone in regulating body growth performance.

Selenium is the cofactor of many kinds of proteins and enzymes, such as selenoprotein P and GPx [1, 8]. The enzyme of GPx is required for the protection of the body against oxidative damage from hydrogen peroxide and other lipid hydroperoxides and derivatives [31, 32]. There are four kinds of GPx in the body. Types 1 and 2 of GPx were cytoplasm and plasma [33]. The basal diet contained Se at 0.095–0.128 μg/g of total solids, which satisfied the nutritional needs of the chick (0.100 mg Se/kg of feed dry matter) as set by the National Research Council [20]. Adequate Se is essential for the protection of the immune system from oxidative damage [34]. Dietary Se supplementation holds promise as a means of treating inflammatory conditions, rejuvenating the aging immune system, and protecting the organism from pathogens [8, 35]. When the concentration of Se reaches 0.5 μg/g [36, 37], the activity of plasma GPx (GPx1) is higher than those of groups without supplementation. The activity of GPx2 in the breast muscle with Se supplementation was also significantly increased. Chick supplemented with SEBC had even higher activities of GPx1 and GPx2. Results of other indices of antioxidation, such as the concentration of MDA and activities of SOD and T-AOC, were all improved in the plasma and breast meat with 0.5 μg/g Se and CB supplementation. The antioxidative effect of SECB was optimized for Chinese Huainan Partridge chicks in winter, which are subjected to cold and transport stressors.

Our data indicated that the concentrations of plasma total cholesterol and triglycerides were decreased in chicks treated with compound *Bacillus* and SECB, and did not change in IS- and flavomycin-supplemented chicks. *Bacillus* regulates the metabolism of body lipids [38]. HDLC, mainly synthesized in the liver and the small intestine, plays an important part in eliminating serum cholesterol. We found that supplemental IS and flavomycin play no role in reducing the plasma LDLC levels and increasing HDLC levels, which suggested little effect on lipid metabolism. However, the effect of SECB was obvious. Herein, the main effector was CB, which is in accordance with previous results [38].

When probiotics are used to enhance the meat quality, their effects have been queried, and many opposite results have been reported. Some studies have reported advantages of probiotic supplementation [13, 39, 40], whereas others reported no beneficial effects [24]. L* and b* values correlated positively with drip loss and cooking loss. The reason for the reduced L* and b* values might be associated with the elevated antioxidant activities of CB and SECB, which could protect cells from damage, prevent cell sap extravasation, and reduce the light reflection. Poultry meat is easily attacked by free radicals and oxidized because of a high concentration of polyunsaturated fatty acids [41]. Therefore, the antioxidant condition of the muscles influences the meat quality. Our result indicated that no differences were observed in the L*, a*, and b* values in the IS and flavomycin group compared to those in the control group. However, the L*, a*, and b* values improved in the CB and SECB groups; the effect of SECB was stronger than that of CB. The water-holding capacities, comprising cooking loss and shear force, are also crucial indices of meat quality, because some nutrients are easily lost during exudation by water loss [42]. Shear force is often expressed as the capacity for tenderness, and is one of the crucial sensory qualities that influences the consumer [43]. According to Maltin, tenderness is the most important factor that affects meat acceptance [44]. Our results suggested that water-holding capacity, shear force, and cooking loss were improved with CB and SECB supplementation. However, no effects were observed in the IS and flavomycin groups compared to the control group. The dietary addition of SECB combined the positive effects of Se and *B. subtilis*, and could have strong regulating roles in antioxidation and metabolism in improving meat color.

Previous studies have reported that Se supplementation can increase the Se content of tissues, although the results varied [45]. In our study, the Se contents in the breast meat and blood were very significantly increased in the IS and SECB groups, which indicated that the supplemental Se in SECB had a significant influence on the Se content of breast muscle; this is in accordance with previous reports [29, 37]. The effect of SECB was greater than that of IS in this experiment, suggesting that SECB had greater availability as an Se resource. However, antibiotic supplementation negatively affected the breast meat flavomycin concentration, suggesting that antibiotics should be used judiciously despite its role in infectious disease prevention.

## Conclusions

In conclusion, dietary SECB supplementation improved the growth performance and breast meat quality for the rearing of healthier Chinese Huainan Partridge chicks in cold environments, and could be used as a substitution to antibiotic drugs.

## Abbreviations

a*: Redness
ADG: Average daily weight gain
b*: Yellowness
F/G: Ratio of feed to gain
L*: Lightness
Se: Selenium
SECB: Selenium enriched compound bacillus
WHC: Water-holding capacity
